# The game changing role of traditional ecological knowledge based Agri amendment systems in nutrient dynamics in the stress prone semi arid tropics

**DOI:** 10.1038/s41598-021-88801-8

**Published:** 2021-05-03

**Authors:** Seema B. Sharma, G. A. Thivakaran, Mahesh G. Thakkar

**Affiliations:** 1 Department of Earth and Environmental Science, KSKV Kachchh University, Mundra Road, Kachchh, Bhuj, Gujarat India; 2 Blue Bay Coastal Research Foundation, Chennai, India

**Keywords:** Environmental impact, Ecology, Environmental sciences

## Abstract

The major crop nutrients determine the nutritional content and vigor of crops. The deficiency or occurrence below minimal level of any of the nutrients are often seen as a cause of poor growth or complete crop failure. The present study was an attempt to understand the impact of Traditional Ecological Knowledge (TEK) (A1)vis-à-vis conventional chemical intensive (A2)agriculture amendment systems in altering/modifying the nutrient dynamics of the soil with respect to nitrogen (N), phosphorus (P), potassium (K) and sulphur (S), calcium (Ca), magnesium (Mg) levels in the pre, mid and post-harvest phases of crop in six cropping seasons spread across four years. The study area was a geo-ecologically unique terrain of Kachchh, Western India, a typical representative of allied arid and semi-arid tropics that are prone to various natural threats and stressors like drought, salinity and erratic rainfall pattern that affect the agri-management activities. Seasonal amendment data, clearly depicts that TEK based systems were efficient in soil organic carbon (SOC) accrual over seasons, an important trait required in challenging settings of tropical aridisols. The major primary (N, P, K) and secondary (S, Ca, Mg) nutrients were at par or higher than integrated chemical intensive systems. TEK based amendments ensured proper and timely management of nutrients in the soil. This inherent value addition offered by indigenous manure applications is an important step in climate change mitigation measures and overall agricultural sustainability.

## Introduction

Long-term environmental implications of chemical-based inputs for improving agricultural production have raised several questions^2,34^. A multi-criteria-based approach for understanding agriculture sustainability is indispensable^15^. In recent times, a revival of Traditional Ecological Knowledge (TEK) based agro-ecosystem management strategies is the need of the time. The article 8j of the United Nations Convention on Biological diversity hasrecognised the TEK as knowledge, innovations and practices of indigenous peoples and local communities relevant for the conservation of biological diversity and have undertaken to respect, preserve and promote its wider application^6^. Unfortunately, a copious amount of this knowledge has been lost since the Green Revolution of mid-sixties and existing knowledge remains either unused or under used. The Intergovernmental Panel on Climate Change has emphasised on the role of indigenous knowledge and crop varieties in climate adaptation^25^. The United Nations Sustainable Development Goals (SDG)^38^, including goals 2, 3, 12, 13 and 15 are closely linked to this interest in preservation of indigenous knowledge for sustainable development.

‘Rig Veda’ and ‘Krishi Parashara’ are few of those valuable treatises from ancient Indian scriptures that date back to 400 BC and the soil adaptive management strategies mentioned in them hold relevance till date. Similar indigenous knowledge exists in different parts of the world^30,31^. As stated in the United Nation’s second SDG, food security is a complex condition requiring a holistic approach involving a series of complementary actions that promote sustainable agriculture that is adapted to climate change. Sustainable and cost-effective soil management using local and indigenous resources is also indispensable to reversing land degradation and biodiversity loss (SDG 15). Yet, agricultural policies as well as associated research and development in public sector institutions in developing countries as India continue to primarily emphasize chemical and input intensive farming technology. A parallel strategy is needed to promote the revival of TEK based soil management techniques, encourage innovation and, incentivize the adoption of this knowledge, especially for small and marginal farmers that have not benefitted (economically or socially) from the Green Revolution.

Increasing the production in tune with environmental sustainability is the biggest thrust laid on our agriculture management systems. The world population by 2050 is expected to be 9.7 billion; this increased population pressure would demand an increased food production^40^. However, the most important entity in agricultural production-the land resource, specifically the arable land is limited and its expansion beyond a certain threshold is not possible. Hence, meeting the demands of this growing population would require an increased yield from present arable land. However, this increase in yield has to be beckoned by soil nutrient management, in a way that a self-reliant system is developed that maintains soil fertility in the longer course of time.

The major crop nutrients-nitrogen, phosphorus and potassium along with Sulphur, calcium and magnesium determine the nutritional content and vigor of any crop. The deficiency or occurrence below minimal level of any of these nutrients is often seen as a cause of poor growth or complete crop failure. Although in conjugation external physical factors like water availability, salinity stress or any other natural calamity affect the overall yield but the importance of the nutrients in soil systems is of paramount importance.

Agriculture in the past few decades, specifically the post ‘Green Revolution’ era of 1960′s has already witnessed the detrimental effects of synthetic chemical inputs based nutrient management systems^34^. The chemicals that enter our food system through the chemical fertilizers have crippled our overall ecosystem health that includes the basic production unit (the soil), the producing unit (farming community) and finally the consumer unit (the food chain). We have been ushered into an era of Agriculture 4.0^17^ and it is quite imperative that we realize the importance of an agri-system that can sustain itself as well as the coming generations and management practices that support the ecosystem health and nutrient content of ‘soil’ and the ‘produce’ are now the ‘need’ and not a ‘choice’.

The present study was an attempt to understand the impact of Traditional Ecological Knowledge (TEK) (A1) vis-à-vis conventional chemical intensive (A2) based agriculture amendment systems in altering/modifying the nutrient dynamics of the soil with respect to N, P, K and S, Ca, Mg levels in the pre, mid and post-harvest phases of crop over six cropping seasons spread across four years in a semi-arid stress prone terrain of Kachchh, Western India.

## Materials and methods

### Study site and field selection strategy

**Figure 1:**
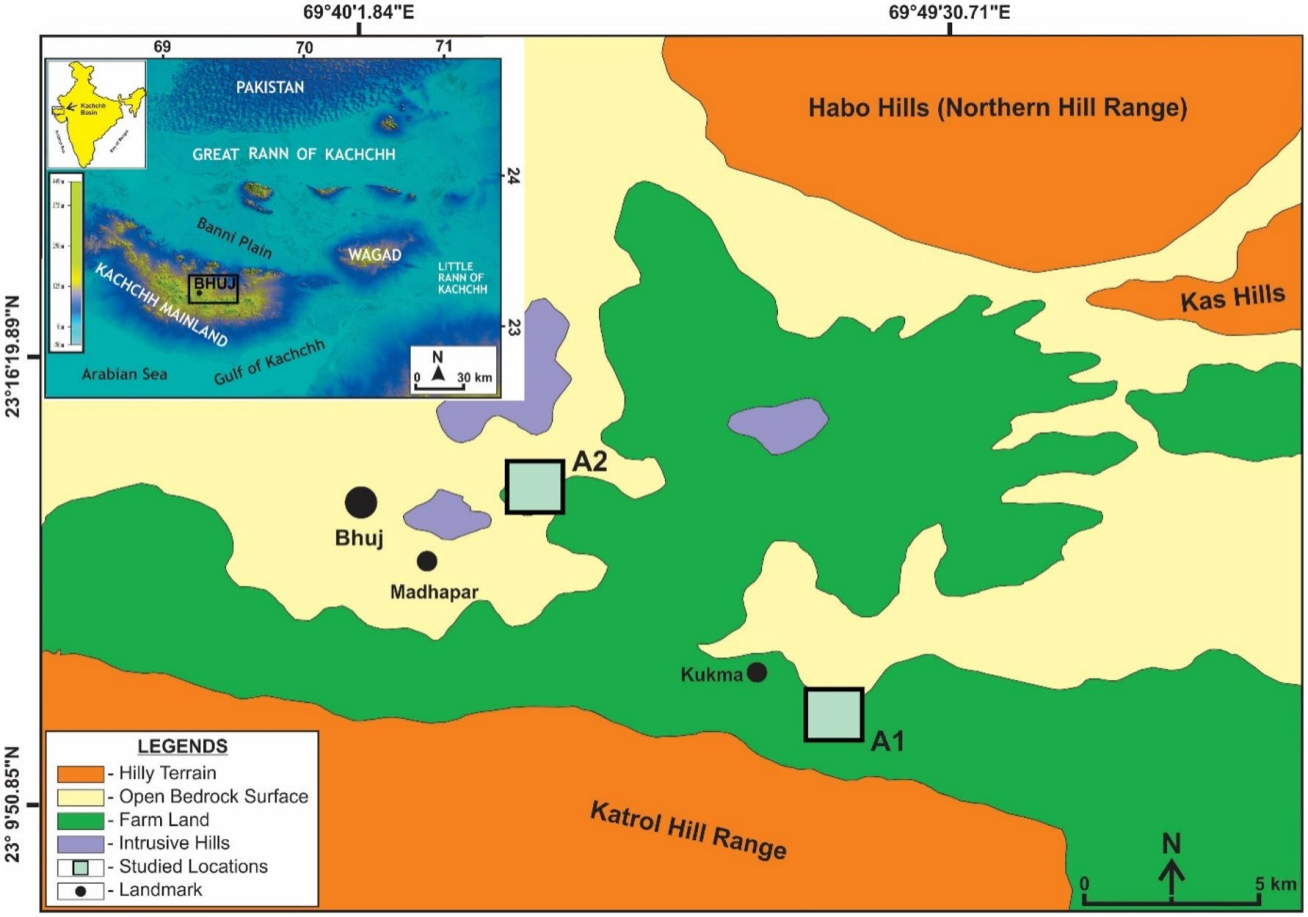
Study area location (generated using coreldraw version X7 and arcGIS version 10.4).

Kachchh district (22° 44′ to 23° 48′ N and 68° 22′ to 71° 02′ E in western most Indian state of Gujarat, is a true representative of arid tropics. Drought, salinity, unpredictable rainfall pattern are the features that alter the agriculture scenario of this zone. The soils in the present study sites belong to great group typic camborthids and are in general calcareous^22^. The upcoming damming projects have altered the rain dependent feature of agri systems here over the past decade^14^. The selected sample sites are geomorphically secured farmlands made on the alluvial formations resting on the Cretaceous fluvial sandstones as bedrocks while the southern Katrol hill range provides the alluvium from the marine Jurassic rocks with higher inherent salinity^19,27^ (Fig. 1).

In our present study we demarcated two agri management systems viz. indigenous knowledge based TEK (A1) and chemical intensive integrated (A2). Ten field per system were chosen and soil sampling was carried out in the pre, mid and post-harvest phases of crop in six cropping seasons spread across four years. In India, cropping pattern follows two distinct seasons: July to October is Kharif season and Rabi season from October to March. For the present study, three Kharif (Summer crop; 2012, 2013, 2014) and three Rabi (winter crop; 2011–2012, 2012–2013, 2013–2014) were studied. For TEK based agri systems farm yard compost (FYC) and ‘*Jivamrit S*’, a fermented concoction consisting of cow urine, cow dung, jaggery, gram flour and soil^24^ were applied. FYC was applied at the rate of 4 ton (Mg) /hectare as a basal dose before sowing and *Jivamrit S* was applied with watering twice, at seven days interval from sowing. This concoction/jivamrit was prepared on site in composting pits. The chemical intensive integrated amended fields used farm yard manure (FYM) as a basal dose (applied at rate of 1 ton/ha) along with synthetic fertilisers as sources of nitrogen and phosphorus. For nitrogen, urea at the rate of 60 kg/ha was applied as top dressing at around 15–20 DAS (days after sowing) and for phosphorus, Di ammonium phosphate (DAP) at the rate of 40–60 kg/ha was applied as top dressing at around 15–20 DAS. Consistency in management practices for both the amendment systems was maintained for all the six cropping seasons studied.

Using standard soil sampling protocols^28^, sample collection was carried out from the rhizosphere of the crop up-to the depth of 12–15 cm. As the experiments were carried out at the farmers’ fields and not experimental stations hence the crops were not restricted to one or two varieties. However, the sites for experiment grew crops viz*.* Maize (*Zea mays*), Sorghum (*Sorghum bicolor*) as Kharif (summer) and wheat (*Triticum aestivum*), castor (*Ricinus communis*) crops as Rabi (winter) depending upon the monsoon which is highly erratic. The fields under study followed monoculture cropping pattern and the system was not highly mechanized except for ploughing, where both the type of fields used tractors. In the pre-sowing phase, mid-phase of crop and post-harvest phase of crop, four samples were collected per ha per field and were further pooled to form one composite sample. These composite samples were then analysed in triplicates. Soil samples were divided into two parts, out of which one part of the sample was air dried in shade and sieved through a sieve size of 2 mm. and analysed for physical and chemical characteristics and the second part stored at 4 °C and analysed for microbiological parameters^42^.

### Soil parameters

The available nitrogen in the soil was determined following alkaline permanganate method^35^. For available phosphorus, Olsen’s method for neutral alkaline soil^23^ was adopted. The total phosphorus in soil samples was extracted by a mixture of concentrated sulphuric acid, hydrofluoric acid and hydrogen peroxide^4^ and the P concentration of the extract was determined using the same method as for available P. The exchangeable potassium was determined in normal neutral ammonium acetate (CH_3_COONH_4_) extract of soil^13^. Sulphur was estimated turbidimetrically using a spectrophotometer^11^. Exchangeable calcium and magnesium were determined in 1 N KCl extracts of soil by titration with EDTA (Ethylene Diamine Tetra Acetic acid)^11^. In^41^ rapid titration method was adopted to determine soil organic carbon (SOC) and organic matter (OM).

### Statistical analysis

For the statistical analysis of obtained datasets, GLM (General Linear Model) three-way analysis of variance using SAS version 9.3 software and SPSS ver. 20 (IBM) and significant effects (P < 0.05) were adopted. Further, using SAS ver. 9.3, a multiple comparison was done among all the main effects and interactions to identify the homogenous effects using Tukey’s HSD (honestly significant difference) comparison for Least Square Means (LSM). The treatments which get same letter grouping are at par and the treatment pairs getting different letter grouping are significantly different. Correlation analyses were carried out to determine the nature and magnitude of relationship between various soil and microbial parameters in different seasons and phases for both the amendments. A probability level of 0.01 was considered to be statistically significant. Pearson’s test of correlation (two tail) was performed. The study area map was generated using coreldraw version X7 and arcGIS version 10.4.

### Ethical approval and consent to participate

NA. Present study was in compliance with local and national regulations.

## Results

### Available major nutrients

#### Available nitrogen

**Figure 2:**
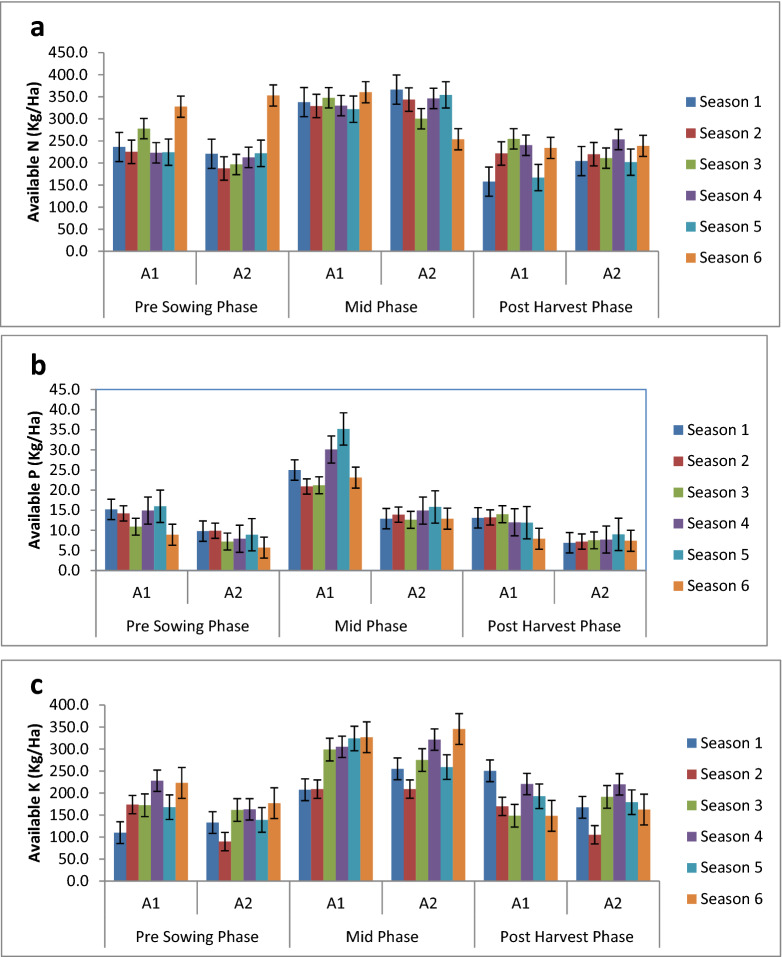

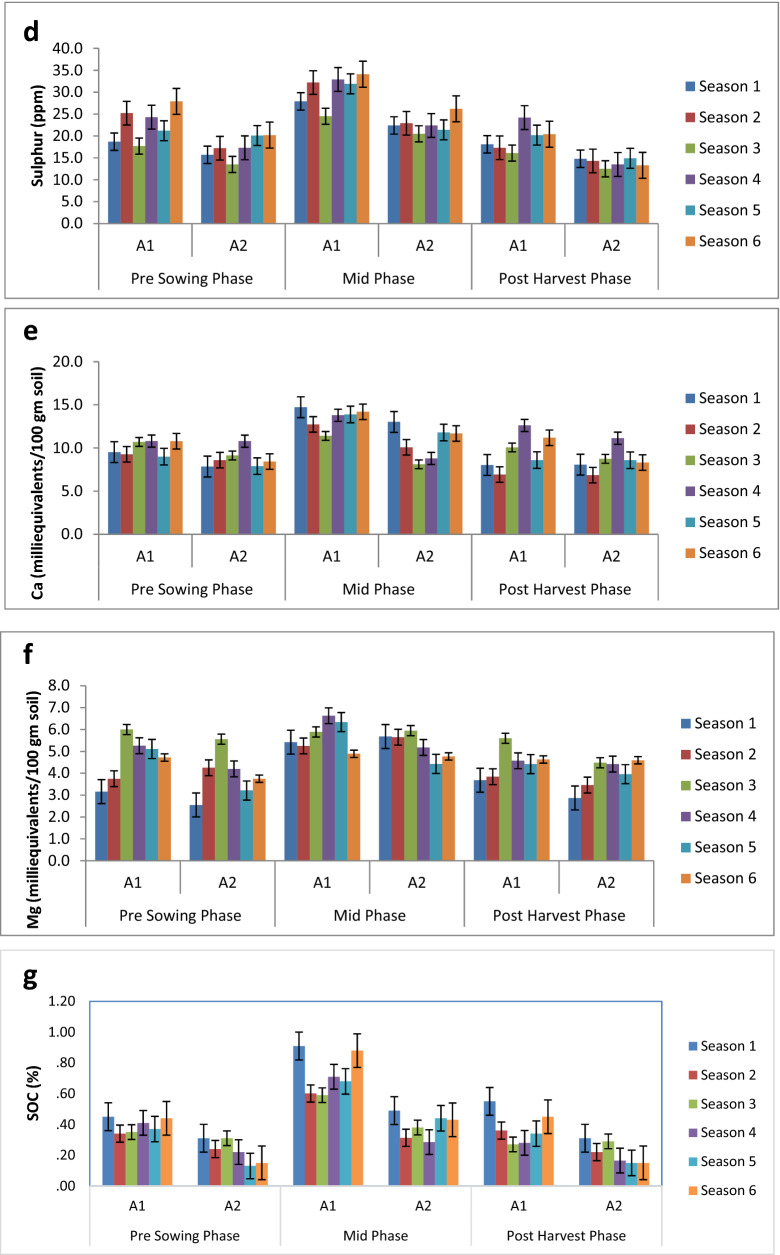
(**a**) Seasonal Variation in Available Nitrogen in A1 and A2 under different phases of crop growth. (**b**) Seasonal Variation in Available P in A1 and A2 under different phases of crop growth. (**c**) Seasonal Variation in Available Potassium in A1 and A2 under different phases of crop growth. (**d**) Seasonal Variation in Sulphur in A1 and A2 under different phases of crop growth. (**e**) Seasonal Variation in Calcium in A1 and A2 under different phases of crop growth. (**f**) Seasonal Variation in Magnesium in A1 and A2 under different phases of crop growth. (**g**) Seasonal Variation in SOC in A1 and A2 under different phases of crop growth.

The range of available nitrogen across the six cropping seasons for amendment A1 and A2 was from 103 kg/ha to 476 kg/ha with SE =  ± 4.5 kg/ha and SD =  ± 85.5 kg/ha (Table 2). There was marginal difference in mean values of available nitrogen in A1 (267.62 kg/ha; SE =  ± 6.21 kg/ha) and A2 (260.38 kg/ha; SE =  ± 6.5 kg/ha). Seasonal variation in available nitrogen in A1 and A2 under different phases of crop growth is depicted in Fig. 2a. The mid-phase had a higher available nitrogen value (332.64 ± 6.6 kg/ha) than both the pre- sowing (242 ± 6.2 kg/ha) and post-harvest phase (217 ± 5.3 kg/ha) across all the amendments and seasons. The highest available nitrogen value amongst the six seasons was observed in season 6 (294.56 ± 11.04 kg/ha) and lowest in season 5 (248 ± 11.04 kg/ha). The GLM (General Linear Model) three-way analysis of variance Table 1 shows that the effect of cropping season and phase of the crop had statistically significant effects on the available nitrogen. Amendment A1 had mean N levels equivalent to 267.62 kg/ha and A2 had 260.38 kg/ha, however it was not significant statistically i.e. there was no significant difference in the means of available nitrogen in the two amendments**** (Table 2). However significant difference in the mean values was observed in six cropping seasons and also in three phases of the crop. The season by amendment interaction was significant (F (5,324) = 3.59, P = 0.0036) and to further understand which interaction affected the most the Tukey’s comparison lines table was analysed which shows that across seasons, season 6 of A1 had highest N (307.43 kg/ha) and least in season 3 in A2 (236.03 kg/ha). The correlation matrix (Table 3) shows a highly significant positive correlation of available N with SOC.

**Table 1:** The F statistics table for dependent variables available N, P, K and SOC using the GLM procedure.

Source	DF	Available N (Kg/Ha)		Available P (Kg/Ha)		Available K (Kg/Ha)		SOC (%)	
		F Value	Sig	F Value	Sig	F Value	Sig	F Value	Sig
Season	5	4.30	0.0008	8.47	< .0001	17.32	< .0001	14..49	< .0001
Phase	2	115.10	< .0001	159.58	< .0001	150.74	< .0001	198.56	< .0001
Amendment	1	1.23	0.2690	182.14	< .0001	9.21	0.0026	199.23	< .0001
Season*phase	10	6.74	< .0001	2.25	0.0024	5.45	< .0001	3.12	0.0017
Season*amendment	5	3.59	0.0036	1.97	0.0585	2.03	0.0742	3.25	0.0017
Phase*amendment	2	1.73	0.1784	19.70	< .0001	2.83	0.0606	33.16	< .0001
Season*phase*amendment	10	1.68	0.0831	2.12	0.0693	3.22	0.0006	1.62	0.0756

Source	DF	Available sulphur ppm		Calcium milliequivalents/100 gm soil		Magnesium milliequivalents/100 gm soil			
		F Value	Sig	F Value	Sig	F Value	Sig		
Season	5	16.72	< .0001	9.21	< .0001	12.88	< .0001		
Phase	2	177.64	< .0001	75.60	< .0001	40.34	< .0001		
Amendment	10	198.53	< .0001	62.37	< .0001	18.43	< .0001		
Season*phase	1	2.13	0.0221	9.68	< .0001	3.14	0.0008		
Season*amendment	5	3.96	0.0017	1.59	0.1620	2.79	0.0175		
Phase*amendment	2	3.97	0.0198	7.89	0.0005	0.47	0.6250		
Season*phase*amendment	10	1.48	0.1454	1.27	0.2458	1.38	0.1872

**Table 2:** Descriptive statistics.

	N	Range	Minimum	Maximum	Mean		Std. deviation
	Statistic	Statistic	Statistic	Statistic	Statistic	Std. error	Statistic
Soc	**360**	**.93**	**.02**	**.98**	**.3821**	**.01075**	**.19402**
Availn	**360**	**373.00**	**103.00**	**476.00**	**264.0065**	**4.50964**	**85.56440**
Availk	**360**	**396.00**	**80.00**	**476.00**	**206.3725**	**4.46447**	**84.70741**
Availp	**360**	**49.00**	**2.00**	**59.00**	**12.96**	**.0.63**	**6.79**
Sulphur	360	36.00	10.00	46.00	21.0528	.37214	7.06082
Calcium	360	14.70	4.30	19.00	10.1671	.15083	2.86174
Magnesium	360	6.70	1.80	8.50	4.6702	.08149	1.54607
Valid N (listwise)	360

**Table 3:** Pearson’s correlation matrix (2-tail) for the SOC, major and minor nutrients.

	SOC (%)	Available N (Kg/Ha)	Available K (Kg/Ha)	Available P (Kg/Ha)	Available Sulphur ppm	Calcium milliequivalents/100 gm soil	Magnesium milliequivalents/100 gm soil
SOC(%)	1	.343**	.378**	.672**	.468**	.447**	.300**
Available N (Kg/Ha)	.343**	1	.359**	.307**	.398**	.351**	.256**
Available K (Kg/Ha)	.378**	.359**	1	.378**	.404**	.337**	.324**
Available P (Kg/Ha)	.67**	.307**	.378**	1	.568**	.381**	.367**
Available Sulphur ppm	.468**	.398**	.404**	.568**	1	.463**	.251**
Calcium milliequivalents/100 gm soil	.447**	.351**	.337**	.381**	.463**	1	.313**
Magnesium milliequivalents/100 gm soil	.300**	.256**	.324**	.367**	.251**	.313**	1

**Correlation is significant at the 0.01 level (2-tailed).

#### Available phosphorus

Amendment A1 had a higher available P value as compared to A2. The mid-phase had a higher available P value (20.02 ± 0.87 kg/ha) than both the pre- sowing (10.96 ± 0.42 kg/ha) and post-harvest phase (10.08 ± 0.32 kg/ha) across all the amendments and seasons. The highest available P value amongst the six seasons was observed in season 5 (16.36 ± 1.4 kg/ha) and lowest in season 6 (11.35 ± 0.91 kg/ha). Seasonal variation in available P in A1 and A2 under different phases of crop growth is depicted in Fig. 2b. Further the two-way interaction studies of season by phase yielded significant F values foravailable P (F (10,324 = 2.25, P < 0.001)) indicate that the effect of the season on available P values is dependent on the crop phase. The correlation matrix (Table 3) shows a highly significant positive correlation of available P with SOC.

#### Available potassium

The range of available K across the six cropping seasons for amendment A1 and A2 was from 80 to 476 kg/ha with SE =  ± 4.46 kg/ha and SD =  ± 84.70 kg/ha (Table 2). However A1 had a higher value (215.31 kg/ha; SE =  ± 6 kg/ha) as compared to A2 (197.42 kg/ha; SE =  ± 6.56 kg/ha). The mid-phase had a higher available K value (267.93 ± 5.12 kg/ha) than both the pre-sowing (161.48 ± 5.12 kg/ha) and post-harvest phase (179.64 ± 5.12 kg/ha) across all the amendments and seasons. The highest available K value amongst the six seasons was observed in season 4 (242.93 ± 7.2 kg/ha) and lowest in season 1 (187.25 ± 7.2 kg/ha). Seasonal variation in available K in A1 and A2 under different phases of crop growth is depicted in Fig. 2c. The statistical data (Table 1) shows that the availability of potassium in the soil was dependent on season (F (5,324 = 17.32, P < 0.0001), phase (F (2,324 = 150.74, P < 0.0001) and amendment (F (1,324 = 9.21, P = 0.002). The two-way interaction of season and phase was also showing statistically significant results; however, the season-amendment and phase-amendment interaction were not significant enough, similarly, the three-way interaction of season, phase and amendment was also not remarkably significant. So the significant interaction of season and phase was further analysed and it was found that mid-phases of all seasons were significantly higher and in particular season 6 mid-phase had highest available K (336.1 ± 12.5 kg/ha) and it was at par with season 3, 4 and 5 but season 1 and 2 was significantly lower in their K values with season 2 having least K values of 208.96 ± 12.5 kg/ha. A comparative study of two amendments shows that available K was substantially higher in A1 (215.31 ± 4.16 kg/ha) than A1 (197.42 ± 4.16 kg/ha). The correlation matrix (Table 3) shows a highly significant positive correlation of available K with SOC.

#### Available sulphur, calcium and magnesium

The range of available S across the six cropping seasons for amendment A1 and A2 was from 10 to 46 ppm with SE =  ± 0.37 ppm and SD =  ± 7.06 ppm (Table 2). However, A1 had a higher value (24.15 ppm; SE =  ± 0.55 ppm) as compared to A2 (17.95 ppm; SE =  ± 0.38 ppm). The mid-phase had a higher available S value (26.60 ± 0.4 ppm) than both the pre- sowing (19.91 ± 0.38 ppm) and post-harvest phase (16.63 ± 0.38 ppm) across all the amendments and seasons. Seasonal variation in available sulphur in A1 and A2 under different phases of crop growth is depicted in Fig. 2d. The highest available S value amongst the six seasons was observed in season 6 (23.68 ± 0.54 ppm) and lowest in season 3 (17.35 ± 0.54 ppm). The range of available Ca across the six cropping seasons for amendment A1 and A2 was from 4.3 milliequivalents/100 g soil to 19 milliequivalents/100 g soil with SE =  ± 0.41 milliequivalents/100 g soil and SD =  ± 7.86 milliequivalents/100 g soil. The mean value for available Ca for both the amendments was 10.16 ± 0.15 milliequivalents/100 g soil. However A1 had a higher value (11.01 milliequivalents/100 g soil; SE =  ± 0.15 milliequivalents/100 g soil) as compared to A2 (9.32 milliequivalents/100 g soil; SE =  ± 0.15 milliequivalents/100 g soil) (Table 2). The mid-phase had a higher available Ca value (12.01 ± 0.18 milliequivalents/100 g soil) than both the pre- sowing (9.39 ± 0.18 milliequivalents/100 g soil) and post-harvest phase (9.09 ± 0.18 milliequivalents/100 g soil) across all the amendments and seasons. The highest available Ca value amongst the six seasons was observed in season 4 (11.31 ± 0.26 milliequivalents/100 g soil) and lowest in season 2,3 and 5 (9.0 ± 0.26 milliequivalents/100 g soil). Seasonal variation in available Ca in A1 and A2 under different phases of crop growth is depicted in Fig. 2e. The range of available Mg across the six cropping seasons for amendment A1 and A2 was from 1.80 milliequivalents/100 g soil to 8.50 milliequivalents/100 g soil with SE =  ± 0.08 milliequivalents/100 g soil and SD =  ± 1.5 milliequivalents/100 g soil (Table 2). The mean value for available Mg for both the amendments was 4.67 ± 0.08 milliequivalents/100 g soil. However A1 and A2 did not differ in mean Mg levels. The mid-phase had a higher available Mg value (5.5 ± 0.11 milliequivalents/100 g soil) than both the pre- sowing (4.2 ± 0.11 milliequivalents/100 g soil) and post-harvest phase (4.2 ± 0.11 milliequivalents/100 g soil) across all the amendments and seasons. The highest available Mg value amongst the six seasons was observed in season 3 (5.5 ± 0.16 milliequivalents/100 g soil) and lowest in season 1 (3.8 ± 0.16 milliequivalents/100 g soil). Seasonal variation in available Mg in A1 and A2 under different phases of crop growth is depicted in Fig. 2f. SOC has shown positive significant correlation with Available S, Ca and Mg (Table 3).

#### Soil organic carbon

The range of SOC across the six cropping seasons for amendment A1 and A2 was from 0.02% to 0.98% with SE =  ± 0.01% and SD =  ± 0.20% (Table 2). However, A1 had a higher value (0.47%; SE =  ± 0.01%) as compared to A2 (0.29%; SE =  ± 0.01%). The mid-phasehad a higher SOC value than both the pre- sowing and post-harvest phase across all the amendments and seasons. The highest SOC value amongst the six seasons was observed in season 1 (0.47 ± 0.02%) and lowest in season 5 (0.32 ± 0.03%). Seasonal variation in SOC in A1 and A2 under different phases of crop growth is depicted in Fig. 2g.

Tukey’s HSD test was applied to assess the pair-wise significance of the means of each of the factors and their interactions. Interaction effects for SOC revealed that all the interactions viz. season by amendment (F (5,324) = 3.25, P < 0.001, season by phase (F (10,324) = 3.12, P < 0.001),) and phase by amendment (F (2,324) = 33.16, P < 0.0001) are significant.

Cropping seasons affected by drought Season 2 (Kharif 2012) and season 3 (Rabi 2012–2013) had higher SOC levels in A1 (0.44% and 0.45% in season 2 and 3, respectively) than A2 (0.24% and 0.34% in season 2 and 3 respectively) that clearly points to the idea that organic inputs provide better resilience towards drought.

## Discussion

Plant growth depends on nutrients availability from soil which has to be supplied by appropriate use of fertilizers. Nitrogen is a major nutrient for sustaining food production in the tropical soils^16^. With soil nitrogen system being a dynamic one, its content varies widely depending on the environment. The data results show that the effect of cropping season and cropping phase had statistically significant effects on the available nitrogen. However, there was no significant difference in the means of available nitrogen in the two amendments. This can be attributed to slow release of nutrients from organic matter. In case of amendment A2, available N levels were probably maintained due to inputs of nitrogenous fertilizer urea. The insignificant variation within amendments could be due to fixation of atmospheric nitrogen by the root nodules present in root system of the crop^21^. However, significant difference in the mean values was observed in six cropping seasons and also in three phases of the crop. Higher values of available N in the mid-phase of crop are perhaps because this is the crop phase which receives maximum external fertilization and also the fertilisers applied during the pre-sowing phase are carried over in this mid-phase as well^39^. Overall, the amendment systems did not affect the N values as much as they did for other nutrients. This is in contrast to other findings. Bellakki and Badanur^3^ observed an increase in available nitrogen due to organic materials application, due to the direct addition of nitrogen through organic materials and greater multiplication of soil microbes, which convert organically bound nitrogen to inorganic form.

Phosphorus is the second major nutrient required for plant growth. Major percentage of total soil phosphorus is in a locked form and hence rendered available only when it is solubilised and mineralised to bio-available forms. Phosphorus deficiency in Indian soils is highly widespread and majority of soils are not able to furnish sufficient quantities of phosphorus on a sustained basis^37^. However, phosphate solubilizing microbes are found in higher numbers in organic manures and compost^31–34^. The applied synthetic forms of P fertilizers like DAP (Di Ammonium Phosphate) provide available P but soon it gets fixed and is rendered useless to the crops. The Kachchh ecoregion has high soil total P as depicted by our study. As evident from interaction studies, there was a significant effect of phase, season, and amendment on the total P and available P levels. Variations in soil P fractions over seasons have been observed by other workers^9^ and^20^. The phase statistics have confirmed that during mid-phase available P was higher than post-harvest and pre-sowing phases. This concludes that the solubilisation and mineralization of organic and inorganic fractions of total P occurs and it is rendered useful to crops in a bio available form though microbial-activities, specifically at the crucial mid-phases of crop cycle^34^. Across seasons, amendment A1 had significantly higher available P and lower total P than A2. Mineralization and immobilization of phosphorus in soil with the addition of organic source have been reported^7,12^. Incorporation of FYM was found to have a beneficial effect on available phosphorus status of soil^1^. High total P in A2 and higher available P in organic amendments (A1) is in affirmation by workers Sugiyama et al.^36^, Lawanprasert et al.^18^ and Freitas et al.^8^.

Potassium (K) is the third major crop growth nutrient. It exists in soil in different forms and these forms are in equilibrium with each other^10^. The major portion of soil K exists as a part of mineral structure and in a fixed form^26^. The results show that the cropping season, phase of the crop and the type of amendment practices used, all affected the available K values of the soil and the interaction statistics show that the effect of season on the available K depended on the cropping phase but it was independent of the amendment and vice versa holds true. A comparative study of two amendments shows that available K was substantially higher in A1 (215.31 ± 4.16 kg/ha) than A2 (197.42 ± 4.16 kg/ha). Similar results have been shown by Lawanprasert et al.^18^, Reganold et al.^29^ and Sugiyama et al.^36^.

Organic amendments provide advantages beyond the benefits of increased organic matter content on soil chemical properties since nutrients that are seldom applied by farmers (e.g. manganese, zinc, and sulphur) are added as insurance against potential yield limitations. Furthermore, nutrients that are normally applied in commercial fertilizers (e.g. potassium) and liming sources (i.e. calcium, magnesium) are supplemented in organic amendments and permitted to accrue in the soil. The statistically analysed data shows that that the cropping season, phase of the crop and the type of amendment practices used, all three affected the available sulphur, calcium and magnesium content of the soil (P < 0.0001). Increase in sulphur, calcium and magnesium content due to organic inputs has been demonstrated by Bulluck et al.^5^, Lawanprasert et al.^18^, Reganold et al.^29^.

Drought resilience is an added advantage provided by the TEK based systems^32^. In our present study, seasons 2 and 3 were drought affected. However, an interesting observation that came out was that amidst the drought induced stressors, the TEK emerged as adaptable systems. The SOC content and primary major and minor nutrients were at par or higher than integrated chemical intensive systems (Tukeys HSD test, supplementary data attached).

SOC is one of the most important indicators of soil quality; it is directly related to the mineralization of organic matter, and nutrient availability. It could be discerned from the present study that, the effect of the crop phase, cropping season, and the kind of amendments on the SOC of the soil, was highly significant (P < 0.0001). The organic amendments had substantially higher levels of SOC (as compared to chemical based farming systems). This was quite an obvious observation owing to the fact that higher inputs of the organic manures in A1 contribute to more of carbonaceous material which tends to increase the organic content of the soil. The significant positive correlation of SOC with both primary and secondary major nutrients has affirmed the fact that amendments from organic sources provide superior nutrient pools to the soil and even during drought phases the resilience provided is an added advantage. The phase and season statistics data have clearly shown that the organic systems that were managed on the basis of Traditional Ecological Knowledge (TEK) are at par and in many aspects better than the conventional/modern chemical intensive systems. Beyond the benefits of providing increased organic matter content in the soil, the TEK based amendments were also able to insure proper and timely management of major primary (nitrogen, phosphorus, potassium) and secondary (sulphur, calcium and magnesium) nutrients.

## Summary and conclusion

The higher content of soil organic matter and organic carbon in organically amended soils with a higher level of major nutrients, both primary and secondary, have confirmed the concept that organic amendments are able to provide advantages beyond the benefits of increased soil organic matter content that in turn affect soil nutrient dynamics. The seasonal data of SOC clearly shows that TEK based systems were efficient in SOC accrual over seasons, a very important trait required in challenging settings of tropical aridisols. In TEK based systems livestock rearing is a part of agriculture activities and this livestock serves as a source of organic manures applied in the form of various concoctions and processes. In India, the enormous wealth of traditional knowledge can be traced to ancient ‘Vedic’ era. This inherent value addition offered by indigenous manure applications is an important step in climate change mitigation measures. The indigenous knowledge based rich cultural heritage in the agriculture sector of Asia, Australia, Africa, America, and many other parts of the globe is an immense storehouse of adaptive methodologies and systems that are suited to the microenvironment, and yet hold promising chances of replicability in similar climatic zones.

## Supplementary Information

Supplementary Information
