# Supplementary material for: The game changing role of traditional ecological knowledge based Agri amendment systems in nutrient dynamics in the stress prone semi arid tropics
Source: Sci Rep. 2021 May 3;11:9425. doi: 10.1038/s41598-021-88801-8 (PMC8093288; doi:10.1038/s41598-021-88801-8)
Supplement: Supplementary file 1 — Supplementary Information [file 41598_2021_88801_MOESM1_ESM.docx]

**Supplementary data**

**The game changing role of traditional ecological knowledge based Agri amendment systems in nutrient dynamics in the stress prone semi arid tropics**

**Seema B. Sharma^1*^, G. A. Thivakaran^2^ and Mahesh G. Thakkar^1^**

1^*^Department of Earth and Environmental Science, KSKV Kachchh University, Mundra Road, Bhuj, Kachchh, Gujarat, India. Email: [seemabhargavsharma@gmail.com](mailto:seemabhargavsharma@gmail.com),

Orcid ID: <https://orcid.org/0000-0001-9361-5570>

2. Blue Bay Coastal Research Foundation, Chennai, India

**Soil organic carbon (SOC)**

| **Source** | **DF** | **Type III SS** | **Mean Square** | **F Value** | **Pr > F** |
| --- | --- | --- | --- | --- | --- |
| **SEASON** | 5 | 1.00785333 | 0.20157067 | 14..49 | <.0001 |
| **PHASE** | 2 | 5.25811167 | 2.62905583 | 198.56 | <.0001 |
| **SEASON*PHASE** | 10 | 0.37347500 | 0.03734750 | 3.12 | 0.0019 |
| **AMENDMENT** | 1 | 2.75625000 | 2.75625000 | 199.23 | <.0001 |
| **SEASON*AMENDMENT** | 5 | 0.25789000 | 0.05157800 | 3.25 | 0.0017 |
| **PHASE*AMENDMENT** | 2 | 0.85708500 | 0.42854250 | 33.16 | <.0001 |
| **SEASON*PHASE*AMENDME** | 10 | 0.22319500 | 0.02231950 | 1.62 | 0.0756 |

Available N

| **Source** | **DF** | **Type III SS** | **Mean Square** | **F Value** | **Pr > F** |
| --- | --- | --- | --- | --- | --- |
| **SEASON** | 5 | 82795.2300 | 16559.0460 | 4.30 | 0.0008 |
| **PHASE** | 2 | 886357.4561 | 443178.7281 | 115.10 | <.0001 |
| **SEASON*PHASE** | 10 | 259697.0627 | 25969.7063 | 6.74 | <.0001 |
| **AMENDMENT** | 1 | 4721.0598 | 4721.0598 | 1.23 | 0.2690 |
| **SEASON*AMENDMENT** | 5 | 69081.5432 | 13816.3086 | 3.59 | 0.0036 |
| **PHASE*AMENDMENT** | 2 | 13343.9234 | 6671.9617 | 1.73 | 0.1784 |
| **SEASON*PHASE*AMENDME** | 10 | 64862.9710 | 6486.2971 | 1.68 | 0.0831 |

Avail K

| **Source** | **DF** | **Type III SS** | **Mean Square** | **F Value** | **Pr > F** |
| --- | --- | --- | --- | --- | --- |
| **SEASON** | 5 | 270769.3798 | 54153.8760 | 17.32 | <.0001 |
| **PHASE** | 2 | 942826.4404 | 471413.2202 | 150.74 | <.0001 |
| **SEASON*PHASE** | 10 | 170321.9057 | 17032.1906 | 5.45 | <.0001 |
| **AMENDMENT** | 1 | 28795.0292 | 28795.0292 | 9.21 | 0.0026 |
| **SEASON*AMENDMENT** | 5 | 31736.3509 | 6347.2702 | 2.03 | 0.0742 |
| **PHASE*AMENDMENT** | 2 | 17687.2352 | 8843.6176 | 2.83 | 0.0606 |
| **SEASON*PHASE*AMENDME** | 10 | 100563.1075 | 10056.3108 | 3.22 | 0.0006 |

Avail P

| **Source** | **DF** | **Type III SS** | **Mean Square** | **F Value** | **Pr > F** |
| --- | --- | --- | --- | --- | --- |
| **SEASON** | 5 | 989.356467 | 197.871293 | 8.47 | <.0001 |
| **PHASE** | 2 | 7341.867195 | 3670.933598 | 159.58 | <.0001 |
| **SEASON*PHASE** | 10 | 639.711308 | 63.971131 | 2.25 | 0.0024 |
| **AMENDMENT** | 1 | 4240.991068 | 4240.991068 | 182.14 | <.0001 |
| **SEASON*AMENDMENT** | 5 | 246.332672 | 49.266534 | 1.97 | 0.0585 |
| **PHASE*AMENDMENT** | 2 | 1100.511701 | 550.255850 | 19.70 | <.0001 |
| **SEASON*PHASE*AMENDME** | 10 | 399.211169 | 39.921117 | 2.12 | 0.0693 |

Sulphur

| **Source** | **DF** | **Type III SS** | **Mean Square** | **F Value** | **Pr > F** |
| --- | --- | --- | --- | --- | --- |
| **SEASON** | 5 | 1459.780556 | 291.956111 | 16.72 | <.0001 |
| **PHASE** | 2 | 6202.372222 | 3101.186111 | 177.64 | <.0001 |
| **SEASON*PHASE** | 10 | 371.394444 | 37.139444 | 2.13 | 0.0221 |
| **AMENDMENT** | 1 | 3465.802778 | 3465.802778 | 198.53 | <.0001 |
| **SEASON*AMENDMENT** | 5 | 345.380556 | 69.076111 | 3.96 | 0.0017 |
| **PHASE*AMENDMENT** | 2 | 138.605556 | 69.302778 | 3.97 | 0.0198 |
| **SEASON*PHASE*AMENDME** | 10 | 258.361111 | 25.836111 | 1.48 | 0.1454 |

Calcium

| **Source** | **DF** | **Type III SS** | **Mean Square** | **F Value** | **Pr > F** |
| --- | --- | --- | --- | --- | --- |
| **SEASON** | 5 | 188.8525014 | 37.7705003 | 9.21 | <.0001 |
| **PHASE** | 2 | 620.1026939 | 310.0513469 | 75.60 | <.0001 |
| **SEASON*PHASE** | 10 | 397.0389028 | 39.7038903 | 9.68 | <.0001 |
| **AMENDMENT** | 1 | 255.7830625 | 255.7830625 | 62.37 | <.0001 |
| **SEASON*AMENDMENT** | 5 | 32.6364792 | 6.5272958 | 1.59 | 0.1620 |
| **PHASE*AMENDMENT** | 2 | 64.6942917 | 32.3471458 | 7.89 | 0.0005 |
| **SEASON*PHASE*AMENDME** | 10 | 52.1222917 | 5.2122292 | 1.27 | 0.2458 |

Magnesium

| **Source** | **DF** | **Type III SS** | **Mean Square** | **F Value** | **Pr > F** |
| --- | --- | --- | --- | --- | --- |
| **SEASON** | 5 | 100.9566056 | 20.1913211 | 12.88 | <.0001 |
| **PHASE** | 2 | 126.4228772 | 63.2114386 | 40.34 | <.0001 |
| **SEASON*PHASE** | 10 | 49.1364694 | 4.9136469 | 3.14 | 0.0008 |
| **AMENDMENT** | 1 | 28.8886678 | 28.8886678 | 18.43 | <.0001 |
| **SEASON*AMENDMENT** | 5 | 21.8564889 | 4.3712978 | 2.79 | 0.0175 |
| **PHASE*AMENDMENT** | 2 | 1.4751606 | 0.7375803 | 0.47 | 0.6250 |
| **SEASON*PHASE*AMENDME** | 10 | 21.6550528 | 2.1655053 | 1.38 | 0.1872 |
